# Immune checkpoint inhibitor-induced thyroiditis and its potential mechanisms

**DOI:** 10.3389/fendo.2025.1584675

**Published:** 2025-06-04

**Authors:** Xueqian Mao, Chaoming Mao, Jiameng Liu, Xi Wang, Yufei Mao

**Affiliations:** ^1^ Department of Nuclear Medicine, The Affiliated Hospital of Jiangsu University, Zhenjiang, China; ^2^ Department of Ultrasound Medicine, The Affiliated Hospital of Jiangsu University, Zhenjiang, China

**Keywords:** immune-related adverse events, immune checkpoint inhibitors, immune checkpoint inhibitor-induced thyroiditis, thyrotoxicosis, hypothyroidism

## Abstract

The expanding clinical utilization of immune checkpoint inhibitors (ICIs) in oncology has brought increasing attention to thyroid dysfunction as a prominent immune-related adverse event (irAE). Elucidating the pathophysiological mechanisms underlying ICI-induced thyroiditis represents a critical step toward developing evidence-based diagnostic protocols and targeted therapeutic interventions for cancer patients undergoing immunotherapy. This comprehensive review systematically examines current advances in understanding the etiopathogenesis of ICI-induced thyroiditis. First, we described pharmacological characterization of ICIs, then discussed multifactorial analysis of cellular and molecular contributors to thyroid autoimmunity following ICI administration and finally analyzed critical evaluation of emerging hypotheses regarding primary pathogenic drivers. Through this review, we aim to establish mechanistic connections between ICI pharmacodynamics and thyroid tissue immunopathology.

## Introduction

**Figure 1:**
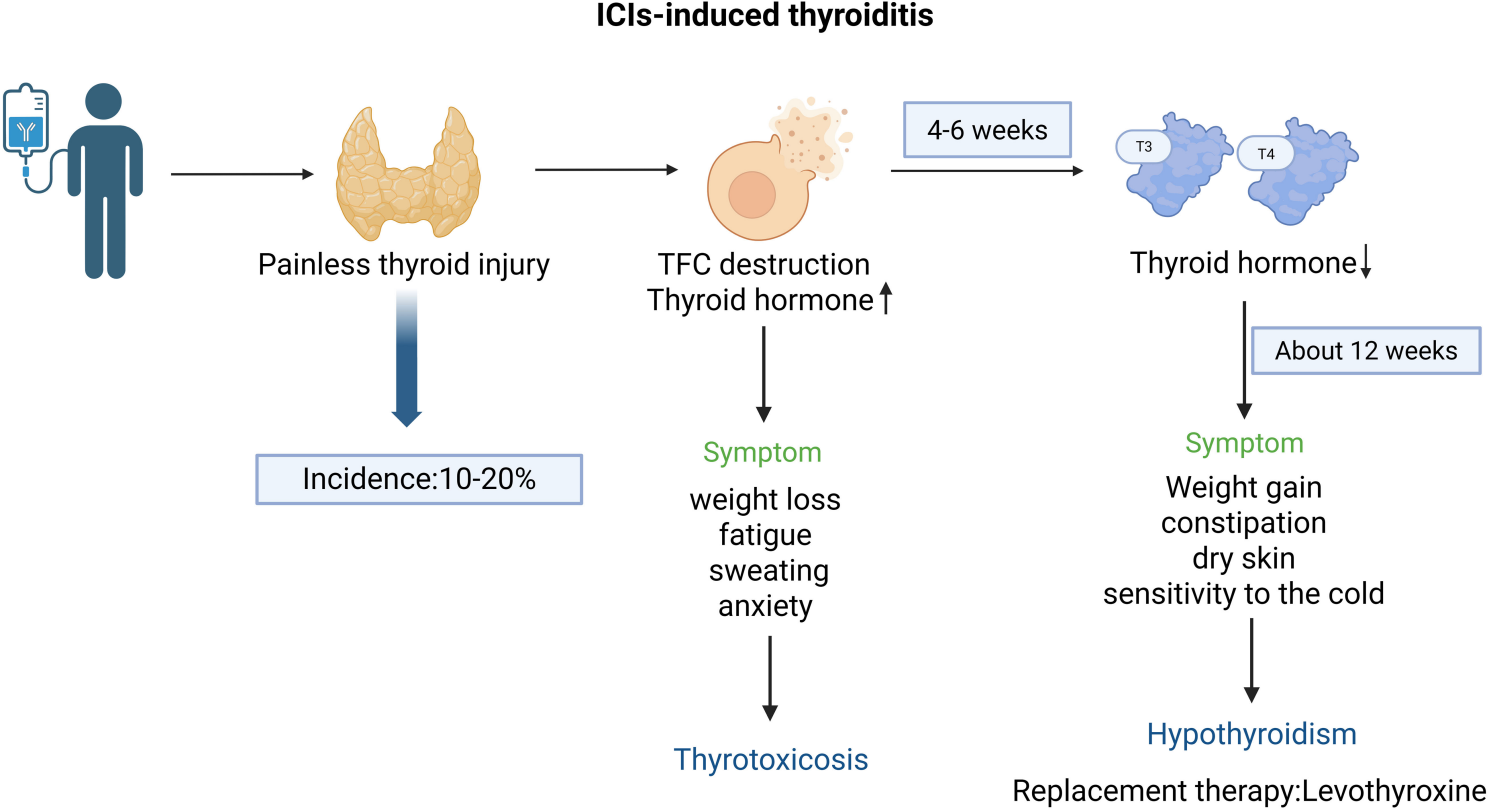
Clinical features and incidence of immune checkpoint inhibitor (ICI)-induced thyroiditis. The clinical features of ICI-induced thyroiditis are thyrotoxicosis caused by destruction of thyroid follicular cells (TFC), followed by a decline in thyroid hormones and development of hypothyroidism.

The therapeutic integration of immune checkpoint inhibitors (ICIs) in oncology has transformed clinical outcomes for diverse malignancies through enhanced T-cell-mediated antitumor immunity via blockade of inhibitory checkpoints including programmed cell death protein-1 (PD-1)/PD-1 ligand (PD-L1) and cytotoxic T-lymphocyte-associated protein 4 (CTLA-4). Paradoxically, this immunomodulatory efficacy coincides with the breakdown of peripheral tolerance mechanisms, manifesting as multi-organ immune-related adverse events (irAEs) (1, 2). Current studies reveal differential irAE profiles demonstrating tumour-type specificity, temporal dependency on treatment duration, and immune microenvironmental modulation patterns (3). Clinically significant irAEs predominantly affect immune-privileged organs including the integumentary system, endocrine axes, gastrointestinal tract, pulmonary parenchyma, and hepatic tissue (4, 5). Particularly in patients treated with ICIs, the incidence of thyroiditis ranges from 10% to 20% (6). The clinical features of ICI-induced thyroiditis are predominantly characterized by destructive thyroiditis, which can lead to transient thyrotoxicosis, followed by the development of hypothyroidism (5, 7). The pathogenesis of thyroiditis may be related to the immune system’s attack on thyroid tissue, which is often associated with the loss of immune tolerance induced by ICIs. Thus, exploring the underlying mechanism of ICI-induced thyroiditis is of particular importance. Through analyzing the characteristics of the effects of ICIs on the immune system, the mechanisms of the autoimmune tendency of the thyroid gland, and the impacts of immune cells, immune molecules, and genetic factors on thyroiditis, we can provide a new theoretical basis and therapeutic guidance for clinical practice. This will enable better management of the side effects induced by ICIs. Clinically, ICI therapy should be discontinued only when symptomatic thyrotoxicosis is present, and long-term levothyroxine replacement therapy should be initiated for persistent hypothyroidism (8) (**Figure 1**).

## Characteristics of the ICIs and irAEs

Immune checkpoints play a crucial role in the immune system by regulating antigen recognition by the T-cell receptor (TCR). These checkpoints encompass both inhibitory and stimulatory immune checkpoint molecules (9, 10). They modulate the intensity of the immune response and uphold immune tolerance, thereby safeguarding normal tissues from the detrimental effects of the immune response (11). During the process of tumor immune escape, inhibitory immune checkpoints have particularly emerged as the focal point of cancer immunotherapy research. Common inhibitory immune checkpoints include PD-1, PD-L1, and CTLA-4 targets (12).

PD-1 is a type I transmembrane protein that belongs to the CD28 immunoglobulin superfamily. It is mainly expressed on tumor-infiltrating activated T lymphocytes, B lymphocytes, natural killer cells, and other immune cells (13). PD-L1 is broadly expressed on tumor cells, antigen-presenting cells (APC), and stromal cells within the tumor microenvironment, with its expression regulated by Interferon-gamma (IFN-γ). In contrast, PD-L2 is predominantly expressed in dendritic cells (DCs) and pulmonary macrophages (14). The interaction between PD-1 and PD-L1 effectively inhibits T-cell activation and it can even induce apoptosis of T-cells and reduce cytokine production, thereby enabling tumor cells to evade immune surveillance (15). Through the use of PD-(L)1 blockers, the anti-tumor function of immune cells can be restored, and the immune escape of tumor cells can be circumvented (16).

CTLA-4 (CD152), a homologue of CD28, mainly exerts its function during T-cell activation (17, 18). CTLA-4 participates in the regulation of immune responses by downregulating the activity of CD4^+^ T effector cells and enhancing the function of regulatory T (T_reg_) cells (19). In the initial phase of T-cell activation, naive T cells bind to major histocompatibility complex (MHC) molecules on APC via the TCR. Meanwhile, CTLA-4 binds to CD80 and CD86 ligands on APC with a higher affinity than CD28 does, thereby inhibiting the activation signal of T cells (20). Blocking CTLA-4 in the tumor microenvironment potentiates T-cell activation, leading to stronger anti-tumor responses driven by CD8^+^ T cells (19, 21).

The blockade of PD-1 and CTLA-4 demonstrates significant differences in the autoimmune phenotype. CTLA-4 inhibitors primarily act in the early stage of the immune response, influencing the initiation phase of T cells in the lymph nodes. In contrast, PD-(L)1 inhibitors function in the effector phase (22). Inhibition of the PD-1 pathway typically leads to alterations in effector cell properties, presenting as a mild and chronic autoimmune phenotype. Conversely, inhibition of the CTLA-4 pathway triggers both intrinsic changes in effector cells and extrinsic changes in Foxp3^+^ T cells, resulting in a more severe non-specific autoimmune phenotype (23). This variability has been validated in irAEs generated by ICI treatment of tumors. Thyroiditis can be induced by both combination therapies (PD-1 inhibitors combined with CTLA-4 inhibitors) and monotherapies (PD-(L)1 inhibitors). The incidence rate is highest with combination therapy and lowest with CTLA-4 inhibitors (5, 6, 24), This suggests that differences in the mechanism of action of ICIs are associated with the varied incidence of ICI-induced thyroiditis. This raises an important question: why does the thyroid gland demonstrate particular vulnerability to immune checkpoint blockade? While the preceding discussion outlines the pharmacological basis of ICIs, the following analysis would elucidate the unique immunological characteristics of thyroid tissue that make it susceptible to autoimmune attack during checkpoint inhibition.

## Common mechanisms of thyroid initiating autoimmune tendency

As a crucial endocrine organ, the thyroid gland exhibits a high incidence in irAEs and autoimmune diseases, including Hashimoto’s thyroiditis (HT) and Graves’ disease (GD). While there is no single definitive explanatory mechanism for its susceptibility, multiple factors interact to contribute to the development of thyroid autoimmunity. The following section examines three intrinsic characteristics of thyroid tissue that predispose it to autoimmune dysregulation during ICI therapy.

### Thyroid-specific proteins as autoantigens

Functionally specific proteins in thyroid tissues, such as the thyroid-stimulating hormone receptor (TSHR), thyroid peroxidase (TPO), and thyroglobulin (Tg), possess unique biological characteristics, such as size, abundance, membrane-binding properties, glycosylation patterns, and polymorphisms, which may disrupt immunological tolerance (25). Clinical observations indicate that when the thyroid’s physiological structural barriers are disrupted by physicochemical factors or infection, function-specific proteins may be released in an “ectopic” manner. This abnormal release can then trigger the autoimmune response. Although the inherently limited central tolerance mechanisms typically eliminate most autoreactive cells, peripheral tolerance remains critical for maintaining immune protection in these organs, as evidenced by studies in animal models and humans with autoimmune disorders (26).

### Immunological sensitivity and tertiary lymphoid structures

Thyroid tissue demonstrates a high level of immunological sensitivity. The observed effects may partly stem from antigen-driven tertiary lymphoid structures (TLSs) that emerge and sustain within pathological microenvironments (27, 28). TLSs are lymphoid aggregates that form postnatally in inflamed, infected, or neoplastic tissues (29). The formation of TLSs depends on the presence of antigens, and their development and persistence are driven by antigen exposure, with subsequent resolution upon antigen clearance (30). Currently, TLSs have garnered significant attention in tumor progression, immune therapy response (31) and irAEs (32). Studies have shown that ICIs can induce and enhance TLSs functionality (33). Although this intervention may improve tumor progression, it could simultaneously heighten immune sensitivity in thyroid tissue.

### Role of thyroid cells in innate immunity

Thyroid follicular cells (TFC) function not only as key effectors of endocrine activity but also as active participants in innate immunity. TFC recognize and react to pathogen-associated molecular patterns (PAMPs) via pattern recognition receptors (PRRs) and also respond to damage-associated molecular patterns (DAMPs) resulting from cellular injury. In an autoimmune context, the response of thyroid cells to these patterns can trigger an up-regulation of inflammatory cytokines such as tumor necrosis factor-alpha (TNF-α), Interleukin(IL)-1, and IL-6, which promote specific immune responses (34). Moreover, TFC which are common targets of autoimmune attacks, also exhibit APC-like cell functionality (35, 36). In the study of autoimmune thyroid disease (AITD), cytokines such as IFN-γ induce TFC to express MHC-II molecules, which present thyroid autoantigens to CD4^+^ T cells, thereby breaking the immune tolerance of the host (37). Recent single-cell transcriptomic studies of GD have revealed that TFC abnormally express a full set of human leukocyte antigen (HLA) molecules, along with elevated expression of CD40 (36). CD40 expression is a characteristic feature of APC (38). However, TFC lack expression of the key costimulatory molecules CD80 and CD86, which are required for T cell activation (36).

## Mechanisms of ICI-induced immune-associated thyroiditis

**Figure 2:**
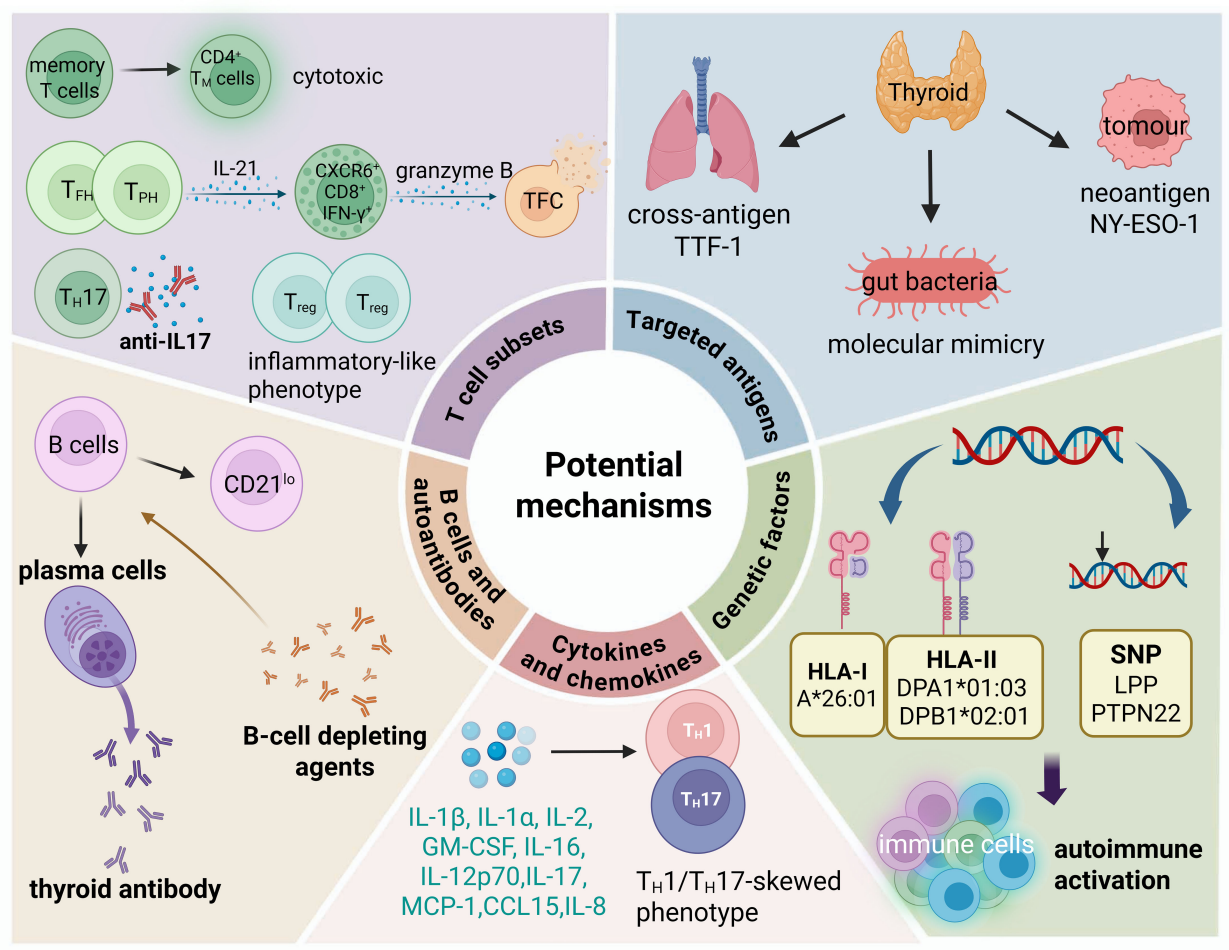
Possible pathogenic mechanisms of ICI-induced thyroiditis. Potential mechanisms include shared antigens, activation and expansion of memory T cells and effector T cells, B cells and autoantibodies, cytokine and chemokine activity, and genetic susceptibility.

Although cancer immunotherapy has become a major focus of research, the underlying mechanisms responsible for ICI-induced thyroiditis remain inadequately elucidated (39). The cytopathology of ICI-induced thyroiditis exhibits distinct characteristics, such as large clusters of necrotic cells, lymphocytes, and CD163^+^ histiocytes (40). The rapid onset of ICI-induced thyroiditis, which differs markedly from typical cases like HT, suggests that the mechanism underlying ICI-induced thyroiditis is multifaceted and intricate. It involves multiple components of the immune system, including antigenic cross-reactivity, T-cell subsets and B cell activation, cytokine and chemokine activity and genetic susceptibility (**Figure 2**).

### Targeted antigens

The initiation of specific autoimmune responses in irAEs is mediated by T-cell clones that recognize cross-antigens present in both normal and tumor tissues (41, 42). For instance, the existence of cross-antigens encoded by the MYH6 gene between cardiomyocytes and tumor cells serves as an autoantigen targeted by CD8^+^ T cells in ICI-induced myocarditis (43). Berner et al. (44) compared peripheral blood mononuclear cells (PBMCs), tumor biopsy specimens, and biopsies from sites of immune-associated skin toxicity in non-small cell lung cancer patients treated with anti-PD-1 through T-cell receptor sequencing (TCRseq). They identified nine identical T-cell antigens shared by tumor tissues and skin, which were capable of stimulating CD8^+^ and CD4^+^ T cells *in vitro* (44). In tissue TCRseq studies of colitis and pneumonia irAEs, identical T-cell clones were detected at the lesion site of the irAEs and at the site of tumor origin (45, 46). However, there are few reports on ICI-induced thyroiditis-related antigens. One study found that in non-small cell lung cancer (NSCLC) patients treated with anti-PD-1 therapy, the expression of thyroid transcription factor-1 (TTF-1) in tumor tissue has been significantly correlated with both the incidence of ICI-induced thyroiditis and the clinical efficacy of ICI therapy (47). TTF-1 is a protein that is widely expressed in the lung and thyroid (48). The findings suggest that TTF-1 may function as a shared antigen between thyroid and lung cancer tissues, potentially contributing to the dual regulation of antitumor immune responses and thyroid autoimmunity. However, the observed association between TTF-1 expression and the incidence of ICI-induced thyroiditis has not yet been definitively attributed to antigenic cross-reactivity. Therefore, further investigation is needed to validate TTF-1 as a potential cross-reactive antigen.

Cancer neoantigens are aberrant peptide fragments arising from tumor-specific mutations that can be recognized by the immune system, thereby eliciting antigen-specific T cell responses directed against early-stage tumors. These neoantigens are detected by the immune system, initiating a tumor-specific immune response, and serve as pivotal targets in the development of cancer immunotherapeutic strategies (48–50). Notably, the potent anti-tumor immunity driven by neoantigens may be accompanied by the risk of autoimmune damage. Vita et al. (51) reported a case involving a 32-year-old female patient with synovial sarcoma who developed GD following administration of the NY-ESO-1 cancer vaccine. This case revealed that multiple epitope regions of the tumor neoantigen NY-ESO-1 exhibit sequence homology with thyroid autoantigens, including TSHR, Tg and TPO, suggesting a mechanism of cross-reactivity (51). Such cross-reactive immune responses may result in T cells targeting both tumor and thyroid tissues. Moreover, following the release of T cell inhibition by ICIs, neoantigen-activated T cells may initiate recognition of additional sequestered thyroid antigens through epitope spreading (49). Thus, the overlap between tumor neoantigens and thyroid autoantigens, coupled with ICI-driven immune activation, provides a plausible mechanism underlying immune-related thyroid dysfunction in cancer immunotherapy.

Emerging evidence indicates that dysregulation of the gut microbiota plays a pivotal role in the development of autoimmune diseases (52). ICI changes immune microenvironment of gut, consequently disrupt the gut microbial community, which can lead to the overactivation of both innate and adaptive immune responses in local tissues, ultimately resulting in systemic immune imbalance (53). Several mechanisms have been proposed to explain the gut microbiota–thyroid axis dysregulation, including molecular mimicry, microbial translocation due to compromised gut barrier integrity, and immune modulation triggered by microbial metabolites (53). Among these, molecular mimicry is increasingly recognized as a critical factor linking gut microbiota to autoimmune dysregulation (53). This mechanism involves structural similarities between microbial peptides and host antigens, which may lead to cross-reactive immune responses. Studies have suggested that vaccines and commensal microbes may influence the development of irAEs through molecular mimicry and related pathways (54). Associations between molecular mimicry and irAEs have been reported in multiple organ systems, including the heart and gastrointestinal tract (55). In a study exploring the mechanisms of gut microbiota’s effects on lung cancer, it was discovered that molecular mimicry might contribute to the development of irAEs and the efficacy of ICIs (54). Notably, certain strains of *Lactobacillus* and *Bifidobacterium* have been shown to induce the production of antibodies that cross-react with thyroid-specific antigens such as TPO and Tg, implicating microbial mimicry in thyroid autoimmunity (56, 57). Similarly, *Yersinia enterocolitica* in small intestinal colitis also exhibits molecular mimicry and may promote the generation and maturation of antibodies against the TSHR (58). Collectively, these findings suggest the overlap between gut microbiota and abundant autoimmune antigenic epitopes may play a part in the onset of irAEs.

The mechanisms of cross-antigenicity and molecular mimicry are involved in ICI-induced thyroiditis remains unclear. Antigens shared between tumors and healthy tissues are marked by high specificity and strong immunogenicity. Identifying cross-antigens not only aids in predicting autoimmune toxic effects but also uncovers potential targets for novel tumor antigens. Therefore, a key challenge lies in distinguishing neoantigens capable of eliciting potent tumor-specific immune responses from those that avoid inducing immune toxicity. Optimizing this balance is essential for improving the efficacy and safety of ICI therapies.

### T-cell subsets

The over-stimulation of TCR signaling associated with ICIs can disrupt the maintenance of peripheral immune tolerance and trigger the activation of potentially autoreactive T-cell clones (59). The loss of T-cell tolerance and the subsequent expansion of autoreactive T-cell clones are among the mechanisms underlying irAEs (60). Given that ICIs exert their effects by enhancing T-cell activity through modulation of T-cell immune checkpoints, T-cell-mediated mechanisms are considered the principal drivers of irAEs. These mechanisms have been shown to involve the activation of targeted memory T cells, the clonal expansion and effector functions of CD8^+^ T cells, the proliferation of T helper (T_H_)17 cells, and the dysregulation of T_reg_ cells.

### Activation of CD4^+^ memory T cells

Memory T cells (T_M_) can be broadly categorized into three subsets: central memory T (T_CM_) cells, effector memory T (T_EM_) cells, and tissue-resident memory T (T_RM_) cells (61). Despite their significance in immune responses, the role of memory T cells in ICI-induced thyroiditis remains poorly understood, primarily due to the limitations inherent in diagnostic biopsies (62). T_RM_ cells, particularly in mucosal barrier organs such as the colon and skin, can be reactivated upon ICIs, leading to inflammation (62). In a study by Lozano et al. (63), multi-omics analysis of early blood samples from melanoma patients revealed a correlation between the abundance of activated CD4^+^ T_M_ cells and increased TCR diversity with the development of severe irAE. Moreover, PD-1, a checkpoint molecule highly expressed on resting T_M_ cells, appears to play a crucial role in regulating T_M_ cell activation (64). This suggests that ICIs may directly affect T_M_ cells during the early stages of irAEs. Evidence from murine models supports this hypothesis. In these models, administration of PD-1 inhibitors following Tg immunization resulted in the infiltration of granzyme B-expressing CD4^+^ T_EM_ cells within the thyroid, indicating their cytotoxic role in thyroid inflammation (65). Clinically, patients with ICI-induced thyroiditis were found to have significantly elevated levels of CD27^+^ CD4^+^ T_EM_ cells in their PBMCs, compared to those without thyroid involvement (65). These findings collectively highlight the pathogenic role of activated T_M_ cells in driving thyroid autoimmunity and suggest that they may serve as early biomarkers for ICI-induced thyroiditis.

### Cytotoxic activity of CD8^+^ T cells

CD8^+^ T cells are well recognized for their direct cytotoxic activity against target cells, and their expansion and activation play pivotal roles in the development of irAEs, including ICI-induced thyroiditis (66, 67). Recent studies have revealed distinct immunological mechanisms underlying ICI-induced thyroiditis. Single-cell RNA (scRNAseq) sequencing analysis of thyroid specimens from patients with ICI-thyroiditis demonstrated a predominant infiltration of clonally expanded cytotoxic CD8^+^ T cells, particularly a subset characterized by CXCR6 expression (67). These CXCR6^+^CD8^+^ effector T cells exhibited elevated production of IFN-γ and granzyme B, suggesting their crucial role in thyroid tissue destruction (67). Importantly, IL-21, secreted by intrathyroidal T follicular helper (T_FH_) and T peripheral helper (T_PH_) cells, was identified as a key driver of this pathogenic CD8^+^ T cell differentiation (67). *In vitro* experiments confirmed that IL-21 stimulation promoted CD8^+^ T cells to acquire an activated effector phenotype, marked by increased CXCR6 expression, enhanced IFN-γ production, and elevated granzyme B levels, collectively contributing to thyroid toxicity (66). Based on this, it can be hypothesized that CXCR6^+^CD8^+^ T cells may constitute the main subset of effector CD8^+^ T cells that respond to irAEs. Additionally, Wu et al. (68) demonstrated in the *in vitro* experiment part, human normal thyroid cells (NTHY) treated with nivolumab (NIVO) and CD8^+^ T cells were cultured. Through a series of experiments, the key protein AKT1 was screened out, and it was found that NIVO could enhance the immune sensitivity of thyroid cells by downregulating AKT1-SKP2 pathway, thereby promoting the killing of thyroid cells by CD8^+^ T cells (68). However, within the context of this study, the *in vivo* experiment did not verify the killing effect of CD8^+^ T cells. In one of the previously mentioned mouse experiments, pre-depletion of CD4^+^ T cells could completely prevent thyroiditis, while depletion of CD8^+^ T cells could partially prevent thyroiditis. The result indicates that CD4^+^ T cells may assist CD8^+^ T cell activation, highlighting immune collaboration in ICI-induced thyroiditis.

### Pro-inflammatory effect of T_H_17 cells

Recent evidence highlights the critical role of T_H_17 cells and their associated cellular and secreted components in the pathogenesis and progression of AITD and irAEs (69, 70). T_H_17 cells, characterized by their production of IL-17A, are recognized for their pro-inflammatory effects and have been implicated in various autoimmune conditions (71). A scRNAseq analysis of peripheral circulating T cells from patients with tumors treated with ICIs revealed a significant increase in the abundance of CD4^+^ T_H_17 cells in those who developed ICI-induced thyroiditis compared to control patients (70). This finding underscores the association between T_H_17 cell expansion and the development of thyroid autoimmune responses in the context of ICI therapies. In addition to clinical findings, studies in tumor-bearing non-obese diabetic (NOD) mouse models have further supported the involvement of T_H_17 cells in ICI-induced thyroiditis (72). Additionally, targeting the T_H_17 and γδT17 cell axis with interleukin-17A (IL-17A) may reduce irAEs without diminishing the antitumor efficacy of ICIs (72).

Targeting the T_H_17 cell axis, particularly through the inhibition of IL-17A, has emerged as a promising strategy to mitigate irAEs without compromising the efficacy of ICIs in tumor control. In a clinical trial involving stage IV melanoma patients who developed multiple concurrent irAEs, including myocarditis, colitis, and rash, the administration of anti-IL-17A therapy resulted in significant regression of these adverse events, alongside an improvement in patient symptoms (73). Importantly, the use of anti-IL-17A therapy did not impair the antitumor effects of ICIs, demonstrating its potential as an effective approach to managing immune-related toxicities while preserving therapeutic efficacy.

### Dysfunction of T_reg_ cells

The activation and proliferation of autoreactive T cells are considered crucial in the development of irAEs. T_reg_ cells play a central role in suppressing the activation of peripheral autoreactive T cells. They maintain immune homeostasis by expressing immune checkpoints that can inhibit the activation and function of other leukocytes (74, 75). Grigoriou et al. (76) discovered that in the peripheral blood of advanced melanoma patients who developed irAEs (including those with ICI-induced thyroiditis) after anti-PD-1 treatment, CD4^+^CD25^+^CD127^+^ T_reg_ cells are expanded and highly express PD-1 and CTLA-4. Moreover, the peripheral T_reg_ cells exhibit characteristic inflammatory transcriptional products, such as IFNG, STAT1, RORC and STAT3. It is evident that T_reg_ cells exhibit an inflammatory-like phenotype in irAEs. Their stability and immunosuppressive function can be maintained through a feedback increase mechanism. This suggests that T_reg_ cells are intricately involved in the immune response related to irAEs and may be a key factor in modulating the occurrence and development of these adverse events.

### B cells proliferation and autoantibodies production

Beyond T cell-mediated mechanisms, emerging evidence highlights the critical involvement of B cells and autoantibodies in irAEs, positioning them as predictive biomarkers (77–79). Studies have demonstrated that early reductions in circulating B cells, alongside elevations in CD21^lo^ B cells and plasma cells, correlate with irAE development (80). Moreover, the severity of the early decline in B cell counts following treatment is directly correlated with both the duration of toxicity episodes and the maximum toxicity grade (80). CD21^lo^ subset, a unique memory B cell population implicated in plasma cell differentiation, may serve as an early predictor of ICI-induced thyroiditis (81). Consequently, early changes in CD21^lo^ B cells may be able to act as one of the predictors of ICI-induced thyroiditis. The aforementioned scRNA-seq analyses of thyroid tissues from ICI-induced thyroiditis patients revealed infiltrating T_FH_ and T_PH_ cells driving thyroid cell destruction (67). Given the established presence of ectopic GCs in GD and HT (82, 83), it is plausible that similar GC-driven antibody production occurs in ICI-associated thyroid inflammation. Furthermore, clinical trials combining B-cell-depleting agents (e.g., rituximab) with ICIs reported reduced hypothyroidism incidence compared to ICI monotherapy, likely attributable to suppressed plasma cell differentiation and antibody synthesis (84). Therefore, it is evident that B cells can be used as a monitoring indicator for ICI-induced thyroiditis and may represent a novel therapeutic strategy for preventing ICI-induced thyroiditis.

The progression of ICI-induced thyroiditis is often associated with the presence of thyroid autoantibodies, which can manifest in three patterns: pre-existing thyroid autoantibody positivity, the emergence of new antibodies post-treatment, or an increase in antibody levels during treatment. Previous studies have shown that patients receiving PD-1 inhibitors are at higher risk for thyroid disease, particularly when thyroid antibodies (TPOAb and TgAb) are present either at baseline or develop during treatment (2). In a study examining the relationship between antithyroid antibodies and ICI-induced thyroiditis, the authors suggested that the presence or increase in TPOAb and TgAb during treatment could serve as reliable biomarkers for identifying patients at higher risk of thyroiditis (85). Furthermore, Ghosh et al. (86) employed autoantigen microarrays to detect 120 autoantibodies (including thyroid antibodies) in patients with advanced melanoma. They found that patients with fewer baseline autoantibodies experienced earlier onset of irAEs (86). This study diverges from prior research in that the authors suggest patients with higher baseline autoantibodies possess a tolerance mechanism that “protects” the body from ICI toxicity (86). These conflicting results underscore the uncertainty surrounding the role of baseline antibodies in predicting thyroid status. Consequently, further in-depth research is required to clarify this issue.

### Increased secretions of cytokines and chemokines

The interplay between cytokines and chemokines in ICI-induced thyroiditis involves complex signaling cascades that amplify immune cell infiltration and thyroid tissue damage (87, 88). Given that organ-specific irAEs may exhibit distinct cytokine profiles, investigating the cytokine signatures associated with ICI-induced thyroiditis is crucial.

A prospective study that evaluated peripheral blood cytokines and chemokines in patients with advanced malignancies undergoing ICI treatment demonstrated that elevated baseline serum levels of IL-1β, IL-2, and granulocyte-macrophage colony-stimulating factor (GM-CSF), along with early decreases in the levels of IL-8, granulocyte colony-stimulating factor (G-CSF), and monocyte chemoattractant protein-1 (MCP-1), were significantly correlated with the development of ICI-induced thyroiditis (89). Liu et al. (90) discovered that early IL-16, IL-12p70, IL-17, IL-1α and C-C motif chemokine ligand 15 (CCL15) could potentially serve as predictive biomarkers for anti-PD-1-induced thyroiditis. The cytokines involved in these studies (IL-1β, IL-1α, IL-2, GM-CSF, IL-16, IL-12p70, and IL-17) are known to have pro-inflammatory effects in autoimmune diseases (91–97). IL-2, primarily produced by CD4^+^ T cells, plays a key role in promoting T_H_1 polarization (93). IL-16 is a chemokine involved in the recruitment and activation of CD4^+^ T cells at inflammatory sites (98). IL-16 enhances T_H_1 polarization and increases the production of the T_H_1 effector cytokine IFN-γ (99). IL-12p70 is a key cytokine that responds to infection and induces T_H_1 responses (100). It can be inferred that ICI-induced thyroiditis is associated with a T_H_1-skewed immune phenotype, a hypothesis supported by recent studies (90). In contrast, MCP-1, which regulates the polarization of T_H_0 cells toward a T_H_2 phenotype, decreases in the context of ICI-induced thyroiditis, further supporting the T_H_1/T_H_2 imbalance (101). Moreover, IL-1β plays a vital role in the differentiation of T_H_17 cells, which are known to drive inflammation, autoantibody production, and immune tolerance disruption in autoimmune thyroid diseases by secreting cytokines like IL-17 and IL-21 (102). The presence of cytokines and chemokines promoting T_H_1/T_H_2 imbalance, a T_H_17-skewed phenotype and CD8^+^ T effect, may contribute to the development of ICI-induced thyroiditis, as reflected by the observed cytokine profile.

Cytokine inhibitors have transformed the treatment landscape of various autoimmune diseases, underscoring their potential role in suppressing irAEs. Among the most studied cytokine inhibitors are those targeting IL-6, IL-1, IL-17, IL-23, and IL-27 (89). Recent studies in animal models have demonstrated that inhibiting IL-17A can reduce the severity of ICI-induced thyroiditis without compromising the antitumor efficacy of ICIs (72). However, further clinical trials are needed to evaluate the safety and effectiveness of cytokine inhibitors in patients with ICI-induced thyroiditis. It is important to note that the cytokine profile associated with ICI-induced immunotoxicity may differ from that of the tumor microenvironment (TME) (103). Consequently, targeting cytokines involved in irAEs may not necessarily impair ICI-mediated antitumor immunity, as the immune mechanisms driving tumor progression differ from those involved in irAEs.

### Genetic factors

The impact of genetic factors on ICI-induced thyroiditis is complex and multifaceted. Research has shown that the HLA system, a key genetic marker, is associated with the development of irAEs (104–106). Both classical HLA-I and HLA-II genes are frequently implicated as drivers of this association. These molecules play a crucial role in presenting antigenic peptides to T cells, enabling the immune system to differentiate between self and non-self-antigens (107). Akturk et al. (105) conducted an HLA-DR genetic analysis in 132 advanced melanoma patients receiving ICIs and found that specific HLA-DR alleles were linked to the development of certain irAEs. Notably, 50% of patients with hypothyroidism carried the DR8 allele (105). Sasaki et al. (108) performed HLA genotyping on 71 cancer patients treated with ICIs and identified HLA alleles A*26:01, DPA1*01:03, and DPB1*02:01 as being associated with ICI-induced thyroiditis. Specifically, A*26:01 and DPB1*02:01 were unique to the thyroid irAE group, while DPA1*01:03 was common across multiple irAE groups. These findings highlight specific HLA genotypes as key factors in the development of ICI-induced thyroiditis. Although the precise mechanisms by which HLA allele variations contribute to irAEs remain unclear, it is known that certain alleles, such as HLA-DR3, are associated with increased susceptibility to AITD like GD and HT (109–111). The amino acid residues linked to disease risk are typically located in the peptide-binding groove or functional pockets of HLA molecules, suggesting their role in antigen presentation and protein stability (109). Therefore, alleles such as A*26:01, DPA1*01:03, and DPB1*02:01 may facilitate the binding of self-proteins, particularly thyroid-derived peptides, leading to a misperception of thyroid tissue as foreign. This misrecognition can trigger immune responses, resulting in ICI-induced thyroiditis. It is important to note that AITD, including HT and GD, have established genetic susceptibilities, with several key loci involved, including HLA, CTLA-4, PTPN22, TSHR, and FCRL3 (112–114). The overlap in genetic susceptibility between AITD and ICI-induced thyroiditis suggests that the latter is not merely a “side effect” of ICI treatment, but rather an exacerbation of pre-existing autoimmune tendencies under conditions of immune activation.

Single nucleotide polymorphisms (SNPs) are DNA sequence variations that arise from single-nucleotide changes at the genomic level, contributing to individual differences (115). Groha et al. (116) identified an IL-7 SNP associated with an increased risk of irAEs, noting that patients with IL-7 germline variants exhibited greater lymphocyte stability following ICI treatment. This suggests that individuals harboring SNPs linked to abnormal autoimmune activation or disrupted signal transduction pathways may be more prone to localized hyperimmune responses, such as those in the thyroid gland, which could lead to ICI-induced thyroiditis. Khan et al. (117) summarized data from seven Phase III clinical trials involving atezolizumab and chemotherapy, using a genome-wide association study (GWAS) approach to identify shared genetic factors for hypothyroidism. They constructed a polygenic risk score (PRS) for validation, which revealed that risk loci in the intronic region of the LPP gene and the PTPN22 rs2476601 (R620W) missense variant were key contributors (117). According to studies, LPP gene and PTPN22 are known to disrupt immune tolerance by modulating T/B cell receptor signaling (117). Additionally, the results highlighted other critical genes involved in T cell initiation (CTLA4) and activation (CD69) (117). These findings underscore the potential for genetic markers to optimize immunotherapy risk assessment, paving the way for personalized approaches in cancer immunotherapy. However, the molecular mechanisms underlying susceptibility SNPs in ICI-induced thyroiditis remain incompletely understood, necessitating further research to clarify their role in the development of these adverse events.

## Conclusion

Among endocrine glands, the thyroid is the most commonly affected by autoimmune disorders, and during ICI treatment, it is particularly susceptible to toxicity. ICI-induced thyroiditis is a complex immunological process primarily driven by the activation of memory T cells upon ICI exposure, which triggers a cascade of downstream events. This leads to thyroid cell destruction through the recruitment of cytotoxic inflammatory cells and the release of inflammatory mediators. Consequently, the follicular structure of the thyroid is disrupted, and the inflammatory response intensifies. Importantly, ICI-induced thyroiditis is typically irreversible. While levothyroxine replacement therapy can effectively restore thyroid function during the hypothyroid phase, it does not address the underlying immune-mediated thyroid tissue damage nor prevent the thyrotoxic phase. Additionally, lifelong hormone replacement therapy places a considerable burden on patients and does not alleviate the potential severity of acute thyroiditis, such as cardiovascular complications. Therefore, more effective treatment strategies are needed to target the key immune mechanisms underlying ICI-induced thyroiditis and prevent the onset of irAEs.

Based on current insights into the mechanisms underlying ICI-induced thyroiditis, potential intervention strategies can be developed at multiple levels. First, preventive monitoring, including regular testing of thyroid function and autoantibody levels at baseline and during treatment, can facilitate the early identification of high-risk patients. Second, targeting key pathogenic pathways offers a foundation for developing targeted therapies. For example, blocking the IL-17A signaling pathway has been shown to alleviate thyroiditis in mouse models while preserving the anti-tumor effects of ICIs, as demonstrated by the use of anti-IL-17 monoclonal antibodies. Additionally, B-cell depletion therapies, such as rituximab, have shown promise in reducing the incidence of thyroiditis in clinical trials. Further approaches include modulating the gut microbiota to inhibit cross-immune reactions driven by molecular mimicry and utilizing HLA genotype or SNP analysis to identify susceptible populations and optimize ICI treatment regimens. The development of predictive models such as cytokine and chemokine profiles offer a new avenue for personalized interventions. Future research also should focus on understanding microbiome-immune interactions, conducting single-cell multi-omics analyses of dynamic changes in the thyroid microenvironment, and developing novel immune modulators that specifically block thyroid autoantigen presentation. These efforts may lead to precise prevention and treatment strategies for ICI-related thyroid toxicity.
